# Multi-level profiling of the Fmr1 KO rat unveils altered behavioral traits along with aberrant glutamatergic function

**DOI:** 10.1038/s41398-024-02815-0

**Published:** 2024-02-20

**Authors:** George Ntoulas, Charalampos Brakatselos, Gerasimos Nakas, Michail-Zois Asprogerakas, Foteini Delis, Leonidas J. Leontiadis, George Trompoukis, Costas Papatheodoropoulos, Dimitrios Gkikas, Dimitrios Valakos, Giannis Vatsellas, Panagiotis K. Politis, Alexia Polissidis, Katerina Antoniou

**Affiliations:** 1 Department of Pharmacology, Faculty of Medicine, School of Health Sciences University of Ioannina, Ioannina, Greece; 2 Laboratory of Neurophysiology, Department of Medicine, University of Patras, Rion, Greece; 3 Center of Basic Research, Biomedical Research Foundation of the Academy of Athens, Athens, Greece; 4 Center of Clinical, Experimental Surgery and Translational Research, Biomedical Research Foundation of the Academy of Athens, Athens, Greece

**Keywords:** Autism spectrum disorders, Hippocampus

## Abstract

Fragile X syndrome (FXS) is the most common cause of inherited intellectual disabilities and the most prevalent monogenic cause of autism. Although the knockout (KO) of the Fmr1 gene homolog in mice is primarily used for elucidating the neurobiological substrate of FXS, there is limited association of the experimental data with the pathophysiological condition in humans. The use of Fmr1 KO rats offers additional translational validity in this regard. Therefore, we employed a multi-level approach to study the behavioral profile and the glutamatergic and GABAergic neurotransmission status in pathophysiology-associated brain structures of Fmr1 KO rats, including the recordings of evoked and spontaneous field potentials from hippocampal slices, paralleled with next-generation RNA sequencing (RNA-seq). We found that these rats exhibit hyperactivity and cognitive deficits, along with characteristic bidirectional glutamatergic and GABAergic alterations in the prefrontal cortex and the hippocampus. These results are coupled to affected excitability and local inhibitory processes in the hippocampus, along with a specific transcriptional profile, highlighting dysregulated hippocampal network activity in KO rats. Overall, our data provide novel insights concerning the biobehavioral profile of FmR1 KO rats and translationally upscales our understanding on pathophysiology and symptomatology of FXS syndrome.

## Introduction

Fragile X syndrome (FXS), a neurodevelopmental disorder, is the most common cause of inherited intellectual disabilities, accounting for 1–2% of all cases and the most prevalent monogenic cause of autism [1, 2]. Excessive expansion of the CGG repeats (over 200) on the 5’ untranslated region of the Fragile X Messenger Ribonucleoprotein 1 gene (FMR1) leads to abnormal methylation and transcriptional silencing, causing lack or deficiency of the Fragile X messenger ribonucleoprotein (FMRP) [3]. FMRP, a predominantly cytoplasmic protein, binds RNA molecules and acts as a negative regulator of translation [4–7]. Patients with FMRP depletion display autism spectrum disorder (ASD) symptomatology, including hyperactivity, cognitive deficits, lack of social interaction, and epileptic seizures [2].

Animal models of FXS include Fmr1 knockout (KO) gene homolog organisms such as drosophila, zebrafish, mice, and more recently rats [8]. Although Fmr1 KO mice have been the leading model, Fmr1 KO rats offer additional translational validity for elucidating the neurobiological substrate of FXS and the efficacy of novel pharmacological interventions, due to their pronounced behavioral and neurobiological complexity [9]. These advantages can be leveraged towards a deeper understanding of the Fmr1 depletion consequences [10]. Fmr1 KO rats exhibit hyperactivity, cognitive impairment, glutamatergic and synaptic plasticity dysregulations [9]. However, limited information exists connecting behavioral deficits to underlying pathology associated with alterations in neurotransmission, electrophysiology, and gene transcription.

To this end, we undertook a multi-level approach to evaluate the Fmr1 KO rat model, including (1) behavioral experiments to assess motor activity and cognitive function, (2) assessment of glutamatergic and GABAergic status in the prefrontal cortex and the dorsal/ventral hippocampus, (3) ex vivo electrophysiological recordings, and (4) transcriptomic profiling. Overall, our data provides novel information concerning the biobehavioral profile of Fmr1 KO rats. We found that these rats possess several features shared with FXS syndrome, including hyperactivity and cognitive dysfunction, while bidirectional glutamatergic and GABAergic alterations were observed in the prefrontal cortex and the hippocampus. These results are coupled to hippocampal changes in excitability and local inhibitory processes along with a specific transcriptional profile.

## Materials and methods

### Animals

Hemizygous wild-type (WT) females and Fmr1 KO male rats on a Long Evans (LE) background (LE-*Fmr1em2Mcwi*) were purchased from the Medical College of Wisconsin (Watertown Plank Rd, Milwaukee, WI, USA) and crossed to obtain littermates from which WT and KO rats were selected for experiments. Ten-week-old male WT and KO rats (raised in the Animal Facility of the University of Ioannina “EL33-BIOexp01.”) were used. Rats were housed in pairs (47.5 cm length × 20.5 cm height × 27 cm width) in a temperature (21 ± 1 °C) and humidity (55% ± 10%) controlled environment. Food and water were available ad libitum.

All experiments were performed during the light phase of a 12 h light/dark cycle (lights on: 7a.m., off: 7p.m.). All animal experiments followed the standard ethical guidelines (European Communities Directive 86/60-EEC) and were approved by the Institutional Animal Committee of the University of Ioannina (6033).

All animals were handled twice daily for 1 week before the behavioral assessment.

To mitigate the impact of the litter effect, particular attention was given to the assignment of animals to experimental groups. Litters were considered as the primary unit of randomization to avoid litter bias. Rats within each litter were randomly assigned to one of the following experimental groups: (i) 1–2 litters/group for behavioral analysis, (ii) One litter/group for immunoblot and neurochemical analysis, (iii) Two litters/group for electrophysiology, and (iv) One litter/group for transcriptomics [11, 12].

### Genotyping by PCR

Tail tip samples were collected from the rats for genomic DNA isolation. Genomic DNA was isolated from the rat tail tip samples using the Tissue DNA Kit (Omega Tek, D3396-02) following the manufacturer’s instructions. PCR was performed using the PCR Kit (Kappa, KK1015) and the Peltier Thermal Cycler (Bio-Rad) according to the manufacturer’s instructions. The primers used for genotyping were as follows:

Forward primer: GTTTATTTGCTTCTCTGAGGG

Reverse primer: ACCTTTTAAATGGCATAGACCT

The PCR reaction was set up with the isolated genomic DNA as the template, and the expected product sizes were 413 bp for wild-type rats (WT) and 415 bp for Fmr1 KO rats. The PCR products were subjected to digestion using the NEB restriction enzyme RsaI (R0167L) in the respective buffer. The digestion was carried out for 3 h at 37 °C, and PCR products were loaded onto the gel for electrophoresis. The DNA bands were visualized under UV light. The observed band patterns on the gel were compared to the expected band fragments to determine the genotype of the rats.

### Behavioral analysis

All behavioral experiments were performed during the light phase between 9:00 and 17:00. The investigator was blinded through all the experimental procedures. One behavioral test was performed each day with an interval of 4 days between tests in the following order:

#### Motor activity—open field

Motor activity was recorded for 60 min with a computerized activity monitoring system (ENV515, Activity Monitor, version 5; Med Associated Inc., USA) in a transparent, cubic open-field apparatus (40 cm × 40 cm × 40 cm). The first 30 min were used to assess spontaneous motor activity, and the second 30 min, to habituated motor activity. Ambulatory distance was used as a measure of horizontal movement and the frequency of vertical activity was also used as a reflection of exploration, locomotion, and emotionality [13]. Lastly, time spent in the center of the apparatus was measured as an index of anxiety [14].

#### Motor activity habituation

This behavioral procedure consisted of a 30 min open-field session every day for 3 consecutive days to assess habituation to a novel environment [15].

#### Novel object recognition test (NORT)

NORT was used to assess recognition memory as previously described with minor modifications [16]. On the training trial (T1), each rat was placed into the apparatus containing two identical objects (familiar) in two adjacent corners, allowing each rat to explore for 5 min. Sixty minutes later, one of the “familiar” objects was replaced by a novel object (T2 phase). Each rat was placed in the apparatus for 3 min, and the time spent exploring each object was recorded. Two WT rats were excluded from the analysis due to poor performance during the training period T1 (exploration time <10 s). The discrimination index (DI) was calculated as the difference in time spent exploring the novel (N) compared to the familiar (F) object divided by the total time spent exploring both objects (DI = (N − F)/(N + F)).

#### Novel object location test (NOLT)

NOLT was used to assess spatial recognition memory as previously described [15]. Like NORT, the T1 phase consisted of free exploration of two identical objects in two adjacent corners of the apparatus for 5 min. Sixty minutes later, the T2 phase was performed where one of the two identical objects was placed in a novel location (N), while the other remained in the same-familiar position (F), as presented in T1. Three WT rats and one KO were excluded from the analysis due to poor performance during the training period T1, expressed as a lack of motivation to explore the objects (exploration time <10 s). Discrimination between the familiar (F) and novel (N) location of the object was assessed, and the discrimination index (DI) was calculated: DI = (N − F)/(N + F).

### Immunoblot assays

Fmr1 KO and WT rats were euthanized by decapitation under isoflurane anesthesia, their brains were immediately removed for dissection of the prefrontal cortex (PFC), the dorsal hippocampus (D. Hip), and the ventral hippocampus (V. Hip), and analyzed with immunoblotting [17, 18]. Following electrophoresis, proteins were transferred onto a nitrocellulose membrane with a Bio-Rad CriterionTM Blotter with wired electrodes (wet transfer, 100 V for 45 min) according to the molecular weight of each protein tested. The antibodies used: anti-NMDA R1 (GluN1) (D6SB7, 1:1000; Cell Signaling), anti-NMDA R2A (GluN2A) (4205S, 1:1000; Cell Signaling), anti-NMDA R2B (GluN2B) (D8E10, 1:1000; Cell Signaling), anti-AMPA Receptor 1 (GluA1) (D4N9V, 1:4000; Cell Signaling), and anti-AMPA Receptor 2 (GluA2) (E1L8U, 1:4000; Cell Signaling). All samples were standardized with anti-αTubulin (T6199, 1:10,000) (Sigma-Aldrich). Optical densities of relevant immunoreactive bands were quantified on ChemiDoc XRS System (Bio-Rad) controlled by Quantity One Software v4.6.3 (Bio-Rad).

### Neurochemical analysis

Brain tissue homogenates, including frontal cortex, dorsal and ventral hippocampus, were used to measure levels of glutamate (Glu), glutamine (GLN), and Gamma Aminobutyric Acid (GABA). A YL9112 Plus Isocratic HPLC Pump (YOUNG IN Chromass Inc., Korea) coupled with a DECADETM Elite Electrochemical Detector (Antec@Scientific, USA) was used. HypersilTM ODS C18, 250 mm × 10 mm × 5 μm column (Thermo Fisher ScientificTM, Massachusetts, USA) was used with pre-column derivatization, as previously described, with some minor modifications [19]. In brief, the voltage of the working electrode was set at +800 mV, and the mobile phase consisted of acetonitrile (Chem-Lab, Belgium): 100 mM monosodium-phosphate buffer pH 5.5, containing 0.5 mM Na2EDTA and 0.1 M Citric Acid 1-hydrate (PanReac AppliChem, Germany). Samples were initially diluted 1:5 with ddH_2_O, then further diluted with 0.1 M Borax buffer (Sigma-Aldrich, St. Louis, USA), including o-Phthalaldehyde (Sigma-Aldrich), pH 10.4. External standards were used each day to generate the calibration curve. Quantification of Glu, GLN, and GABA was performed by comparison of the area under the curve with that of reference external standards using Clarity Software (Data-Apex, Czech Republic), and cycling rates (GLN/Glu and GLN/GABA) were calculated.

### Electrophysiology

#### Hippocampal slice preparation and electrophysiological recordings

Transverse slices 500-μm thick were prepared from the dorsal and ventral hippocampus [20]. Slices were maintained in an interface recording chamber continuously humidified with a mixed gas consisting of 95% O_2_ and 5%CO_2_ and perfused by standard medium containing (mM): 124 NaCl, 4 KCl, 2 CaCl_2_, 2 MgSO_4_, 26 NaHCO_3_, 1.25 NaH_2_PO_4_, and 10 glucose, pH = 7.4 and temperature 30 ± 0.5 °C. Slices were left for at least to recover prior to stimulation and recording. Following stimulation of Schaffer collaterals, we recorded evoked field potentials consisting of excitatory postsynaptic potentials (fEPSPs) and population spikes (PSs) from the stratum radiatum and the stratum pyramidale, respectively. Input–output curves between stimulation current strength and fEPSP or PS were systematically constructed in each slice. The ratio PS/fEPSP was used to estimate neuronal excitability. Paired-pulse stimulation to estimate the effectiveness of synaptic inhibition was used. fEPSP was quantified by the maximum slope of its initial rising phase and PS was quantified by its amplitude, measured as the length of the projection of the minimum peak on the line connecting the two maxima peaks of the PS waveform. Paired-pulse inhibition was quantified by calculating the ratio between the PS evoked by the second pulse and the PS evoked by the first pulse, i.e., PS2/PS1.

### Transcriptomic analysis

#### RNA isolation and RNA-Seq analysis

Fmr1 KO (*n* = 3) and WT (*n* = 3) rats were euthanized by decapitation under isoflurane anesthesia, and total RNA from hippocampal samples was isolated according to the manufacturer’s protocol using TRIZOL reagent (Thermo Fischer Scientific). RNA-Seq experiments were conducted at the Greek Genome Center (GGC) of the Biomedical Research Foundation of the Academy of Athens (BRFAA). RNA-Seq libraries were prepared with the NEBNext Ultra II Directional RNA Library Prep Kit for Illumina, with 1 μg of total RNA input. Library QC was performed with the Agilent bioanalyzer DNA1000 kit and quantitation with the qubit HS spectrophotometric method. Approximately 25 million 100 bp Single-End reads were generated for each sample. Quality Control was performed at the fastq raw data file for each sample using the “FASTQC” software. FastQ files were aligned to rn6 genome using HISAT2 [21]. Counts were defined using HTSeq htseq-count command with the “intersection non-empty” option [22]. The count files were used as Input for DESeq2 [23]. Normalization was performed with the estimate size factor function followed by Differentially Expressed Genes (DEGs) Analysis. DEGs are defined according to *P* value (*P* < 0.05). Gene set enrichment analysis (GSEA) was conducted using the WebGestalt platform (https://www.webgestalt.org/#) [24–27]. The DEG list obtained from the RNA-Seq analysis was used as input for GSEA. Statistical significance was assessed using appropriate algorithms to determine the enrichment of DEGs in specific gene sets. KEGG pathway database (https://www.genome.jp/kegg/) [28–30] was employed to manually identify genes associated with glutamatergic and GABAergic synapses. The genes involved in glutamatergic and GABAergic synapses were identified by cross-referencing the DEG list with the gene members of the respective pathways.

### Statistical analysis

All values are expressed as mean ± SEM. Statistical analyses were performed using IBM SPSS software by implementing Student’s t-test or two-way repeated-measures ANOVA (similar variance between groups). All comparisons were considered significant, where *P* < 0.05.

## Results

### Behavioral analysis reveals hyperactivity and cognitive deficits in Fmr1 KO rats

#### Increased motor activity in Fmr1 KO rats

**Fig. 1:**
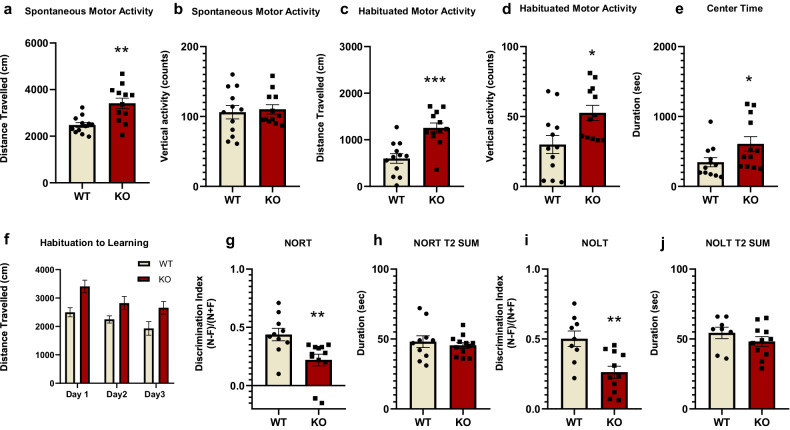
Increased motor activity and impaired recognition and spatial memory for Fmr1 KO rats. Spontaneous and habituated horizontal and vertical motor activity for Fmr1 WT (*n* = 12) and KO (*n* = 12) rats in the open field apparatus. Distance traveled (**a**) and vertical counts (**b**) during spontaneous motor activity. Distance traveled (**c**) and vertical counts (**d**) during habituated motor activity. Time spent in the center of the open-field apparatus (**e**). Habituation to Learning (**f**) of Fmr1 WT and KO rats. Assessment of recognition and spatial short-term memory of Fmr1 WT and KO rats. Discrimination Index (DI) of Fmr1 WT (*n* = 10) and KO rats (*n* = 12) for the Novel Object Recognition Task (NORT) (**g**). Total time spent exploring the two objects during the test phase (T2) of the test (**h**). Discrimination index (DI) of Fmr1 WT (*n* = 9) and KO rats (*n* = 11) for the Novel Object Location Task (NOLT) (**i**). Total time spent exploring the two objects in the test phase (T2) of the test (**j**). All results are represented as means ± SEM; *P < 0.05, **P < 0.01, ***P < 0.001.

The open-field test has been extensively used for measuring spontaneous and habituated motor activity, including horizontal and vertical responses [13, 31]. KO rats showed increased horizontal activity compared to WT rats, both during the first period of spontaneous motor activity (Fig. 1a), as well as the second half, representing habituated motor activity (Fig. 1c). Spontaneous vertical activity was not altered, whereas during the habituated period, Fmr1 KOs demonstrated increased vertical mobility (Fig. 1b, d). KO rats spent statistically significant increased time in the center of the open-field box in comparison to WT (Fig. 1e).

#### Fmr1 KO habituate over time

Open-field habituation over consecutive exposures has been used for the evaluation of non-associative learning and memory [15]. Two-way repeated ANOVA measures did not reveal any statistically significant effect. Overall, both Fmr1 KO and WT rats exhibited decreased horizontal activity over the 3 days of testing and thus, habituated over time (Fig. 1f).

#### Fmr1 KO rats have impaired recognition memory

The NORT assesses short-term recognition memory, and it is a non-rewarding paradigm based on rodents’ spontaneous exploratory behavior [32]. Fmr1 KO rats demonstrated a decrease in the NORT discrimination index, indicating a deficit in recognition memory (Fig. 1g). The total time spent exploring both objects during the test period (T2 SUM) did not show any statistically significant difference between the two groups (Fig. 1h).

#### Fmr1 KO rats exhibited impaired spatial recognition memory

The NOLT is based on the spontaneous exploratory behavior of rodents and is used to assess short-term spatial recognition memory [33]. Fmr1 KO exhibited a lower discrimination index in comparison to their WT counterparts (Fig. 1i), indicating deficits in short-term spatial memory for Fmr1 KO animals. The total time spent during the test period (T2 SUM) did not show any statistically significant difference between the two groups (Fig. 1j).

### Region-specific glutamate receptor expression perturbations in Fmr1 KO rats

Next, we examined potential brain alterations associated with cognitive dysfunction in Fmr1 KO rats and focused on glutamatergic status, including the expression of glutamate receptors (NMDA and AMPA) in the prefrontal cortex and the hippocampus. The GluN2A/2B ratio was also assessed since it provides an index of synaptic activity [34–36]. Taken together, the present results, an opposite status, concerning glutamate protein expression, appeared in the PFC versus the hippocampus in the KO rats.

#### Fmr1 KO rats have increased NMDA receptor GluN2A/2B expression in the PFC and decreased NR2B protein expression in the hippocampus

**Fig. 2:**
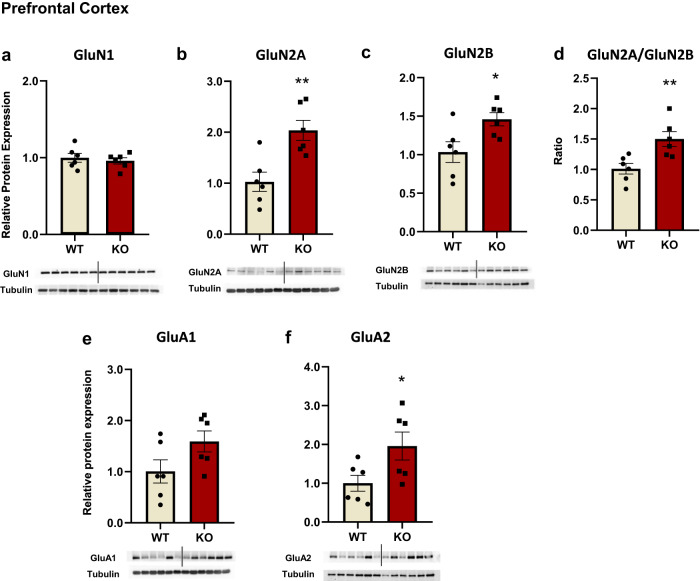
NMDA and AMPA receptors subunits protein expression levels were found elevated in the prefrontal cortex of Fmr1 KO rats. NMDA receptors subunits protein expression levels in the prefrontal cortex of the Fmr1 WT (*n* = 6) and KO (*n* = 6) rats. GluN1 (**a**), GluN2A (**b**), GluN2B (**c**), and ratio of GluN2A/GluN2B (**d**). AMPA receptors subunits protein expression levels in the prefrontal cortex of the Fmr1 WT (*n* = 6) and KO (*n* = 6) rats. GluA1 (**e**) and GluA2 (**f**). The optical density (OD) of each band was divided by the corresponding loading marker. Data are presented as relative protein expression to WT rats. Bellow each graph is presented a representative image from each western blot including a band of the protein of interest and the corresponding loading marker band. All results are represented as means ± SEM; *P < 0.05, **P < 0.01.

In the PFC, GluN1 subunit expression was unchanged, whereas increased GluN2A and GluN2B subunit expression was observed in KO rats (Fig. 2a–c). The GluN2A/GluN2B ratio was also significantly increased in Fmr1 KO rats (Fig. 2d), due to the prominent increased GluN2A protein expression.

**Fig. 3:**
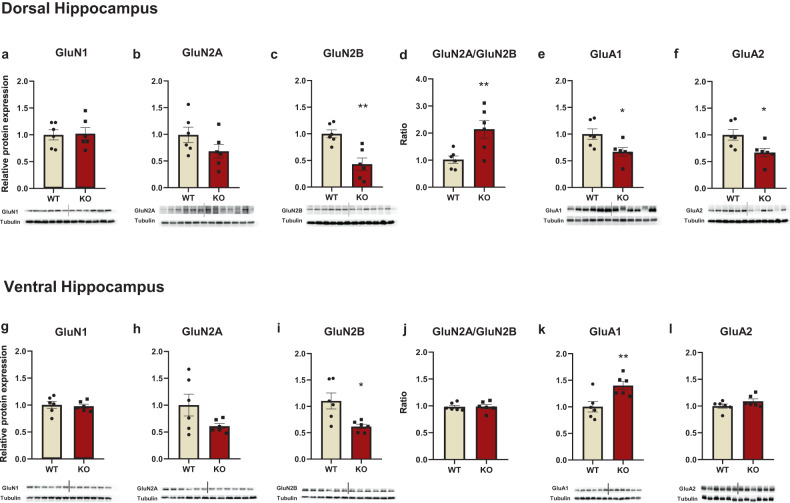
NMDA and AMPA receptor subunit levels were found to be altered in the dorsal and ventral hippocampus of Fmr1 KO rats. NMDA and AMPA receptors subunits protein expression levels in the dorsal and ventral hippocampus of the Fmr1 WT (*n* = 6) and KO (*n* = 6) rats. GluN1(**a**), GluN2A (**b**), GluN2B (**c**), and the ratio of GluN2A/GluN2B (**d**), GluA1 (**e**), and GluA2 (**f**) in the dorsal hippocampus. GluN1 (**g**), GluN2A (**h**), GluN2B (**i**), and the ratio of GluN2A/GluN2B (**j**), GluA1 (**k**), and GluA2 (**l**) in the ventral hippocampus. The optical density (OD) of each band was divided by the corresponding loading marker. Data are presented as relative protein expression of WT rats. Bellow each graph is presented a representative image from each western blot including a band of the protein of interest and the corresponding loading marker band. All results are represented as means ± SEM; *P < 0.05, **P < 0.01.

There was no statistically significant difference in GluN1 levels between WT an KO rats (Fig. 3a, g). In the dorsal hippocampus, GluN2A expression tended toward a decrease (Fig. 3b), while GluN2B expression was significantly lower in the Fmr1 KO rats (Fig. 3c). The ratio GluN2A/GluN2B was significantly increased for Fmr1 KO rats due to decreased GluN2B protein expression levels (Fig. 3d). The same pattern was observed in the ventral hippocampus, however, the GluN2A/GluN2B ratio was similar between both WT and KO rats (Fig. 3h–j).

#### GluA1 and GluA2 expression is altered in the PFC and hippocampus of Fmr1 KO rats

In the prefrontal cortex, AMPA GluA1 subunit expression was unchanged, whereas GluA2 expression was elevated in Fmr1 KO rats (Fig. 2e, f). In the dorsal hippocampus, GluA1 and GluA2 protein expression levels were significantly lower in the KO rats (Fig. 3e, f). In the ventral hippocampus, GluA1 expression was higher in the Fmr1 KO rats (Fig. 3k).

### Region-specific dysregulation of excitatory and inhibitory neurotransmission in Fmr1 KO rats

We next assessed potential regionally distinct alterations in glutamatergic and GABAergic neurotransmitter activity of Fmr1 KO rats.

**Fig. 4:**
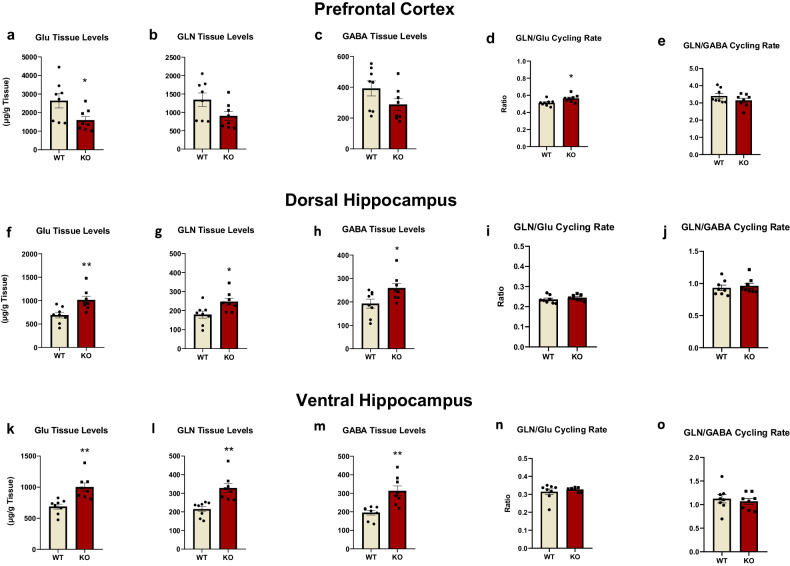
Regionally dependent alterations in glutamatergic and GABAergic neurotransmission activity of Fmr1 KO rats. Glutamate (Glu), Glutamine (GLN), GABA tissue levels and their cycling rates in the prefrontal cortex, the dorsal, and the ventral hippocampus of Fmr1 KO (*n* = 8) and WT (*n* = 8) rats. Glu (**a**), GLN (**b**), and GABA (**c**) along with cycling rate of GLN/Glu (**d**) and GLN/GABA (**e**) in the prefrontal cortex. Glu (**f**), GLN (**g**) and GABA (**h**) along with cycling rate of GLN/Glu (**i**) and GLN/GABA (**j**) in the dorsal hippocampus. Glu (**k**), GLN (**l**) and GABA (**m**) along with cycling rate of GLN/Glu (**n**) and GLN/GABA (**o**) in the ventral hippocampus. All results are represented as means ± SEM; *P < 0.05, **P < 0.01.

Decreased glutamate levels and elevated cycling rate were observed in the PFC (Fig. 4a, d). However, a trend for decreased GABA levels was observed in KO rats.

In the hippocampus, both glutamate and glutamine levels were increased, but the glutamate cycling rate was unchanged (Fig. 4f, g, i, k, l, n). GABA levels were elevated in the dorsal and ventral hippocampus (Fig. 4h, m).

Overall, these neurochemical findings indicate region-specific differential perturbations of neurotransmitter activity consistent with alterations in glutamate receptor expression levels.

### Loss of excitation/inhibition balance in the hippocampus of Fmr1 KO rats

Glutamatergic and GABAergic functions are tightly linked to synaptic transmission and the balance between excitation and inhibition, with the hippocampus being a key brain region for assessing such processes [37].

#### Excitatory synaptic transmission was not different for the KO rats

**Fig. 5:**
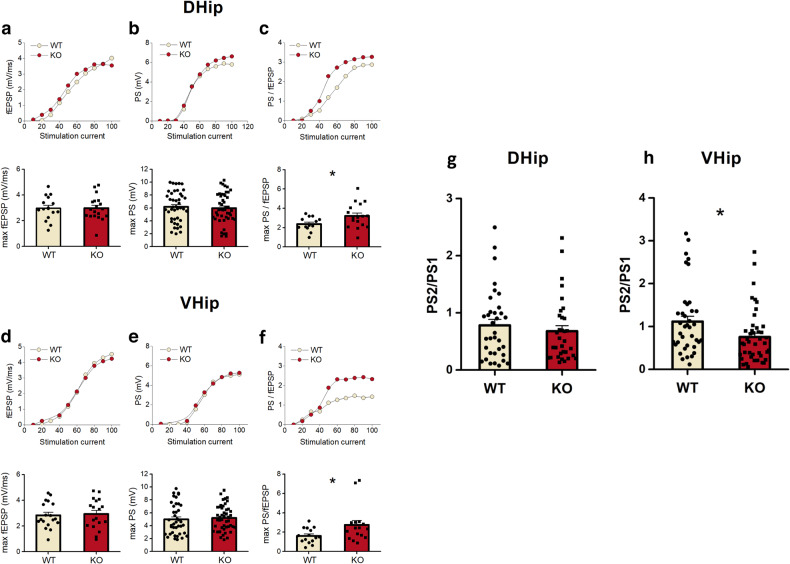
Alterations in excitability and local inhibitory processes in the hippocampus of the Fmr1 KO rats. Comparison of synaptic transmission, neuronal excitation, and neuronal excitability in WT and Fmr1 KO in the dorsal (D. Hip) (**a**–**c**) and ventral hippocampus (V. Hip) (**d**–**f**). Upper graphs in each panel show examples of input–output curves between stimulation current intensity and fEPSP or PS or PS/fEPSP. Synaptic transmission was compared between WT and KO rats by measuring the max fEPSP. Max fEPSP did not significantly differ between the WT and KO rats in either the dorsal (**a**) (WT *n* = 15 slices/12 rats and KO n = 19 slices/19 rats) or the ventral hippocampus (**d**) (WT *n* = 18 slices/16 rats and KO *n* = 18 slices/18 rats). Regarding neuronal excitation we found that max PS did not significantly differ between WT and KO rats in either the dorsal (**b**) (WT *n* = 42 slices/33 rats and KO *n* = 46 slices/35 rats) or the ventral hippocampus (**e**) (WT *n* = 40 slices/33 rats and KO *n* = 46 slices/37 rats). Neuronal excitability was compared between WT and KO rats by measuring the ratio PS/fEPSP at max PS value. Max PS/fEPSP significantly increased in KO compared with WT rats in both the dorsal (**c**) (WT *n* = 13 slices/11 rats and KO *n* = 17 slices/16 rats) and the ventral hippocampus (**f**) (WT *n* = 15 slices/13 rats and KO *n* = 16 slices/13 rats). Paired-pulse inhibition increases in the ventral, not dorsal, KO hippocampus. The effectiveness of paired-pulse inhibition was compared between WT and Fmr1 KO rats by measuring the ratio PS2/PS1 recorded at a stimulation strength that produced a half-maximum PS1. PS2/PS1 in the dorsal hippocampus (**g**) was comparable between WT and KO rats (WT *n* = 34 slices/29 rats and KO *n* = 33 slices/27 rats). However, PS2/PS1 was significantly smaller in KO compared with WT ventral hippocampus (**h**) (WT *n* = 38 slices/30 rats and KO *n* = 44 slices/32 rats), suggesting a higher inhibition in the KO vs WT ventral hippocampus. Collective data are shown in the bottom graphs in each panel and they are represented as means ± SEM; *P < 0.05.

Synaptic transmission and neuronal excitation, respectively, were unchanged in both the dorsal and ventral hippocampus of Fmr1 KO rats (Fig. 5a, d).

#### Maximum neuronal excitation was not different for the KO rats

Neuronal excitation was compared between WT and Fmr1 KO rats by measuring the maximum PS that was not different between them in both dorsal or ventral hippocampus (Fig. 5b, e).

#### Neuronal excitability was higher in the Fmr1 KO rats

Neuronal excitability was subsequently assessed by measuring the ratio PS/fEPSP at the maximum PS value. A significant increase in neuronal excitability was observed in Fmr1 KO rats in both the dorsal and the ventral hippocampus (Fig. 5c, f).

#### Paired-pulse inhibition was higher in the ventral hippocampus of FmR1 KO rats

The effectiveness of paired-pulse inhibition was evaluated by measuring the ratio PS2/PS1 recorded at a stimulation strength that produced a half-maximum PS1.

PS2/PS1 was unchanged in the dorsal hippocampus of Fmr1 KO rats, however, we found that the ratio PS2/PS1 was significantly lower in the ventral hippocampus of Fmr1 KO as compared to WT rats (Fig. 5g, h).

### Transcriptomic analysis: RNA-seq analysis

**Fig. 6:**
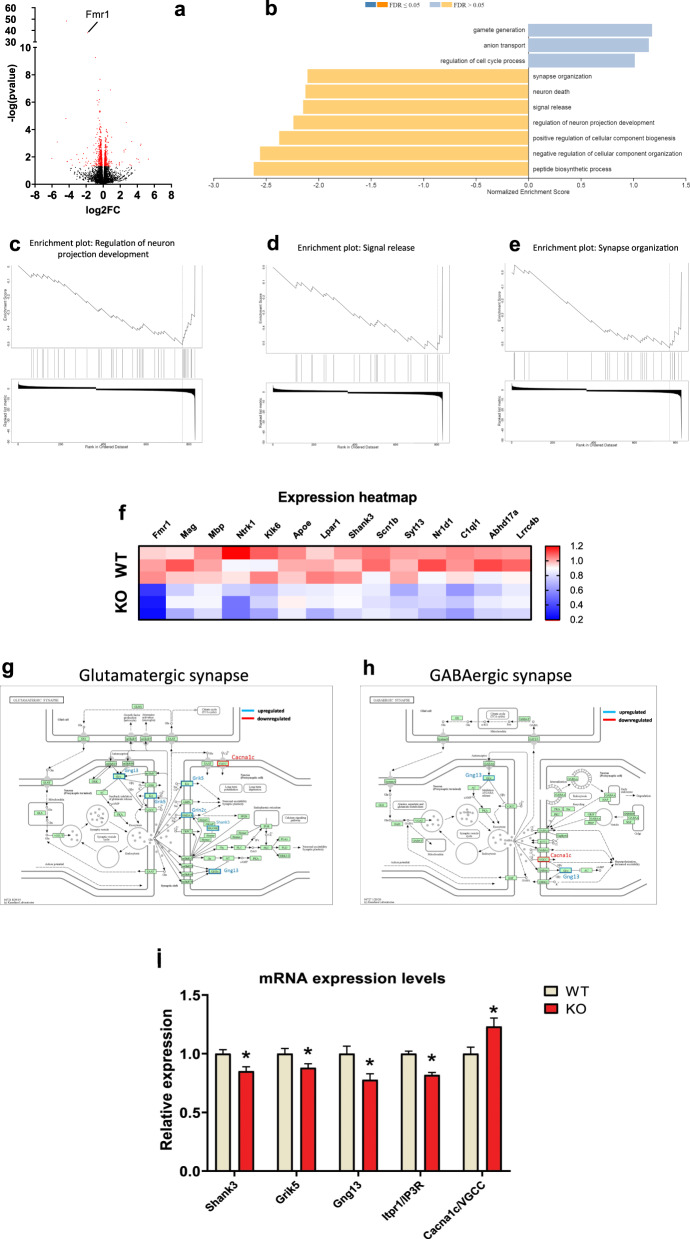
Comprehensive analysis of gene expression and pathway enrichment in the hippocampus of the Fmr1 KO rats. Volcano plot displaying the results of differential gene expression analysis. Differentially Expressed Genes (DEGs) are represented as red dots, while non-DEGs are depicted as black dots (**a**). Histogram presenting the enrichment analysis results from Gene Set Enrichment Analysis (GSEA) performed using the WebGestalt platform (**b**). The histogram illustrates the enrichment of upregulated and downregulated biological processes among the DEGs. **c**–**e** Enrichment plots demonstrating significant enrichment of DEGs in specific biological processes: “Regulation of neuron projection development” (**c**),“Signal release” (**d**), and “Synapse organization” (**e**). Expression heatmap showing gene expression levels of DEGs implicated in the biologic processes mentioned in (**d**–**f**). **g**, **h** Pathway depictions obtained from the KEGG database: Glutamatergic synapse pathway with upregulated genes outlined in red and labeled in red, and downregulated genes outlined in blue and labeled in blue (**g**); GABAergic synapse pathway with upregulated genes outlined in red and labeled in red, and downregulated genes outlined in blue and labeled in blue (**h**). Relative expression levels of the DEGs implicated in both the glutamatergic and GABAergic synapse pathways, allowing for comparative analysis of gene expression changes between the two pathways (**i**).

Our last objective was to employ RNA sequencing (RNA-Seq) in the hippocampus to gain a deeper insight into pathological alterations at the transcription level. The RNA sequencing analysis yielded compelling results, identifying a total of 838 genes that were differentially expressed (Fig. 6a). To further explore the functional implications of these gene expression changes, we performed Gene Set Enrichment Analysis (GSEA) on the identified gene set. This analysis revealed significant enrichments in gene ontologies related to various biological processes (Fig. 6b). Among the downregulated gene ontologies, synapse organization, regulation of neuron projection development, and signal release, were significantly affected in Fmr1 KO rats (Fig. 6b–f). A gene ontology associated with anion transport was also dysregulated in the Fmr1 KO rats, indicating potential disruptions in ion homeostasis and signaling mechanisms (Fig. 6b).

We next performed pathway analysis using the KEGG database. Key pathways involved in neurotransmission and neuronal signaling were significantly dysregulated; specifically, the glutamatergic/GABAergic synapse pathway (Fig. 6g, h). Noteworthy genes, such as *Cacna1c*, encoding a voltage-gated calcium channel subunit, showed upregulation, suggesting impaired glutamatergic and GABAergic synapse function (Fig. 6g, h). Conversely, the downregulation of *Shank3*, involved in synaptic scaffolding and neurotransmitter receptor clustering, indicated disruptions in postsynaptic density organization and synapse function (Fig. 6g). Finally, the downregulation of the *Gng13* gene suggests possible alterations in G protein signaling within the glutamatergic synapse, GABA receptor signaling, and synaptic inhibition (Fig. 6g, h).

## Discussion

Present results demonstrated hyperlocomotion and cognitive deficits for the Fmr1 KO rats. A differentiated glutamatergic and GABAergic profile, in terms of glutamate receptor subunit expression and neurotransmitter activity, between the prefrontal cortex and hippocampus, accompanied the behavioral phenotype. In parallel, an excessive neuronal excitability and lower inhibitory control were observed in the hippocampus of Fmr1 KO rats. These alterations appear to be associated with aberrant gene transcription due to the lack of FMRP that causes downregulation of anion transport, synapse organization, and signal release, showing a potential disruption of synaptic connectivity and neuronal function.

Fmr1 KO rats were hyperactive in a novel environment. This spontaneous hyperactive profile, in terms of horizontal but not vertical activity, indicates a pure motoric activation unaccompanied by pivotal alterations in exploration or emotional state [13, 15, 31]. Hyperactivity was also observed after the habituation period and interestingly, the Fmr1 KO rats remained hyperactive with respect to both horizontal and vertical activity, throughout the habituation period. Noteworthy, both WT and KO rats demonstrated reduced levels of motor activity due to the longer exposure and subsequent familiarity with the open-field apparatus. In agreement with our results, increased motor activity as deduced by the total time spent in the open-field apparatus was observed for Fmr1 KO compared to WT rats [38, 39]. Another study [40] reported that KO rats were hyperactive, only during the first recording interval. On the other hand, previous studies have shown either no change [41–43] or a reduction in spontaneous motor activity compared to the WT counterparts [8, 44]. Repeated exposures to the open field led to a progressive reduction of motor activity for both WT and KO rats, in a similar pattern to the one observed in the first open-field session, indicating unaffected non-associative learning and memory processes for the hyperactive KO rats.

Regarding recognition learning and memory, our results agree with other studies showing deficits in the novel object recognition test [39, 45–47] and the novel location recognition task [48], however, one study did not report impairments in recognition and spatial memory [49].

To summarize our behavioral paradigms have shown that Fmr1 KO rats exhibited hyperlocomotion, a trait that is also observed in most individuals with FXS [50, 51] and most consistent with the profile of most animal models of autism [52, 53]. Lack of any impairment concerning non-associative learning and memory, but the presence of cognitive deficits related to recognition memory indicates the face validity of this FXS model used in the present study. The conflicting results could be attributed to the different rat strains used in these studies and the genetic modification technique applied to the respective strain to develop the Fmr1 KO model.

Analysis of protein expression of specific glutamate receptor subunits revealed a bidirectional expression pattern between the prefrontal cortex and the hippocampus. Concerning NMDA receptors, increased GluN2A and GluN2B subunit expression were observed in the prefrontal cortex while they were reduced or unchanged in the dorsal and ventral hippocampus in Fmr1 KO rats. Interestingly, the GluN2A/GluN2B ratio was elevated in the prefrontal cortex and the dorsal hippocampus of KO rats due to a marked increase in GluN2A expression and decreased GluN2B expression levels, respectively. These results demonstrate for the first time specific and region-dependent changes in the expression of NMDA receptor subunits in an experimental FXS rat model. Experimental studies in Fmr1 KO mice have not shown any differences in GluN2A and GluN2B expression in the PFC [54, 55]. On the contrary, Bostrom et al. (2015), reported a decrease in the protein levels of GluN1, GluN2A, and GluN2B subunits in the prefrontal cortex of KO’s. Regarding the hippocampus, a decrease in GluN1, GluN2A, and GluN2B subunit expression was found in the dentate gyrus of Fmr1 KO mice [56], while other studies observed increased GluN2A and GluN2B expression in both total hippocampal extracts [57] and isolated synaptosomes [58]. The noted discrepancies between our findings and studies in mice may be due to the different species used, the age of the animals tested since the expression pattern of NMDA subunits changes during development, the type of genetic modification (i.e., Zing Finger Nuclease method vs. CRISPR/Cas9) and the tissue preparations used (e.g., whole-tissue extracts or synaptosomes).

According to our findings, the increased GluN2A/GluN2B ratio in the prefrontal cortex and dorsal hippocampus of Fmr1 KO rats provides new evidence of abnormal neuronal activity. This aberrant change in NMDA receptor subunit expression may likely be due to GluN2A being a target protein of FMRP [6, 7] and FMRP’s role as a negative modulator during translation [59, 60]. On the other hand, the decrease in the hippocampal GluN2B levels may be linked to aberrant induction of LTD in Fmr1 KO rats [35], which could potentially explain the cognitive deficits observed as well.

Noteworthy, the NMDA receptor’s function is essential in regulating AMPA recycling and composition in subunits [61]. Therefore, increased levels of GluA2 subunit observed in the prefrontal cortex of Fmr1 KO rats, is likely a compensatory response due to increased levels of NMDARs subunits. This could be also related to disturbances of synaptic plasticity processes due to the channel Ca++ permeability and AMPA receptor membrane recruitment rate [62–64]. Hippocampal changes in AMPA receptor subunit expression appears to be subregion-specific with reduced GluA1 and GluA2 in the dorsal and increased GluA1 in the ventral hippocampus. Studies with Fmr1 KO mice also report alterations in the expression of AMPAR subunits, albeit approaching the hippocampus as a functionally homogenous region. More specifically they have shown a decrease in GluA1 levels in the whole hippocampus [56], or a decrease in GluA2 levels of the hippocampal synaptosomes [65]. Present findings indicate the importance of distinct characterization concerning, AMPA and NMDA receptor composition and function in the different parts of the hippocampus.

The neurochemical analysis showed that the GLN/Glu cycling rate was increased in the prefrontal cortex of KO rats, due to reduced Glu tissue levels, suggesting increased glutamatergic activity. However, hippocampal tissue levels of glutamate, glutamine, and GABA were increased in KO rats that were not accompanied by any alteration in the cycling rates. To date, there are no studies evaluating the levels of these neurotransmitters in Fmr1 KO rats, while studies in mouse models are also limited. In agreement with our findings, reduced glutamate tissue levels were found in the cortex of Fmr1 KO mice [66]. Based on our results and this study, glutamatergic function appears to be dysregulated in Fmr1 KO rats, leading to a hyperglutamatergic state only in the prefrontal cortex. However, GABA tissue levels, like glutamate, depict mostly internal concentration [67] and exhibit a bidirectional pattern between the prefrontal cortex and hippocampus. Interestingly, these differentiated patterns of Glu levels are linked to the alterations in glutamate receptor subunit composition discussed above. Furthermore, disruptions in glutamate and GABA neurotransmission, particularly in the hippocampus, indicate an imbalance of excitation and inhibition upon *Fmr1* deletion, as further supported by our electrophysiology data.

Specifically, Fmr1 KO CA1 apical dendrites displayed unchanged fEPSPs responses after the stimulation of converging Schaffer collaterals compared to WT rats. Additionally, the neuronal excitation of CA1 somata in stratum pyramidale was unaffected, as deduced by the maximum amplitude of PS recorded. These electrophysiology findings could be also associated with the hippocampus’s unchanged glutamate and GABAergic cycling rates. However, KO rats displayed elevated neuronal excitability in both the dorsal and the ventral subdivisions of the hippocampus, in accordance with previous studies [68–71].

Concerning the ventral but not dorsal hippocampus, an interesting finding was the increased effectiveness of the local inhibitory networks since a higher reduction of the second response evoked by two identical electrical pulses applied in rapid succession was observed. This subregion-specific effect can be explained by the well-known structural and functional hippocampal heterogeneity across the dorsoventral axis [72–74]. Hyperexcitability in the dorsal part, the associative subregion of hippocampus alongside the alterations observed in NMDAR and AMPAR subunit composition, may explain the poor recognition performance of Fmr1 KO rats. On the other hand, the hyperexcitability alongside the hyper-responsivity of the ventral hippocampal local inhibitory network could be associated with the unaffected GluN2A/N2B ratio and the increased expression of GluA1 receptors. Taking into consideration, that the ventral part of the hippocampus is more prone to epileptic-like activity [75, 76], it could be suggested that the ventral hippocampal electrophysiological recordings and corresponding neurobiological alterations likely contribute to the development of adaptive neurobiological mechanisms that counterbalance this sensitivity of Fmr1 KO rats.

Finally, RNA sequencing analysis revealed 838 differentially expressed genes in Fmr1 KO rats generally associated with synapse organization, signal release, regulation of neuron projection development, and anion transport. Observed disruptions in the ion transport and signaling pathways could also contribute to hyperexcitability and aberrant synaptic function observed in Fmr1 KO rats. However, other excitation factors should also be taken into account, such as neuromodulators (dopamine, serotonin, and acetylcholine), changes in synaptic plasticity through LTP and LTD, neuroinflammation, epigenetic modifications, and circuit-level interactions [77–79]. Along these lines, pathway analysis revealed substantial dysregulation in the glutamatergic synapse pathway, which provides a compelling link between the altered gene expression and the glutamatergic alterations observed in the Fmr1 KO rats. In parallel to this concept, the upregulation of genes such as *Cacna1c* suggests potential alterations in calcium-dependent signaling processes within the glutamatergic synapse; an effect that is consistent with increased neuronal excitability in Fmr1 KO rats [80]. Interestingly, Cacna1c belongs to the L-type voltage-gated Ca + + channel family, which are targets of FMRP and are found to be dysregulated in FXS animal models [81]. Conversely, the downregulation of *Shank3*, involved in synaptic scaffolding and neurotransmitter receptor clustering, indicates disruption of postsynaptic density organization and glutamatergic and/or GABAergic synapse function. These molecular alterations align with the observed deficits in neurotransmission and subsequent behavioral output [82–84]. Interestingly, the upregulation of the *Cacna1c* gene and the downregulation of the *Gng13* genes contribute to dysregulation of the GABAergic synapse pathway [85] and might be linked to altered inhibitory processes observed in the electrophysiological experiments and the increased GABA tissue content found in the hippocampus.

## Conclusions

Present findings provide evidence linking Fmr1 deletion with cognitive deficits that arise, at least partly, from perturbations of glutamatergic and GABAergic neurotransmission, apparent at the transcription, protein and synaptic level. This complex interplay deregulates glutamatergic transmission, excitation/inhibition balance, and plasticity with relevance to FXS pathophysiology and symptomatology, which is translationally upscaled by the use of a rat model. Importantly, this study highlights the crucial region-dependent nature of these perturbations, providing valuable insights about the circuit and network bases of the pathologies. Further research will strengthen the possibility of identifying new genetic or neuroanatomical targets for the better understanding and treatment of FXS.

## Data Availability

The data that support the findings of this study are available from the corresponding authors upon request.

## References

[1] Song FJ, Barton P, Sleightholme V, Yao GL, Fry-Smith A (2003). Screening for Fragile X syndrome: a literature review and modelling study. Health Technol Assess.

[2] Hagerman RJ, Berry-Kravis E, Hazlett HC, Bailey DB, Moine H, Kooy RF (2017). Fragile X syndrome. Nat Rev Dis Prim.

[3] Zalfa F, Giorgi M, Primerano B, Moro A, Di Penta A, Reis S (2003). The Fragile X syndrome protein FMRP associates with BC1 RNA and regulates the translation of specific mRNAs at synapses. Cell.

[4] Plante I, Provost P (2006). Hypothesis: a role for Fragile X mental retardation protein in mediating and relieving microRNA-guided translational repression?. J Biomed Biotechnol.

[5] Napoli I, Mercaldo V, Boyl PP, Eleuteri B, Zalfa F, De Rubeis S (2008). The Fragile X syndrome protein represses activity-dependent translation through CYFIP1, a new 4E-BP. Cell.

[6] Edbauer D, Neilson JR, Foster KA, Wang C-F, Seeburg DP, Batterton MN (2010). Regulation of synaptic structure and function by FMRP-associated microRNAs miR-125b and miR-132. Neuron.

[7] Darnell JC, Van Driesche SJ, Zhang C, Hung KYS, Mele A, Fraser CE (2011). FMRP stalls ribosomal translocation on mRNAs linked to synaptic function and autism. Cell.

[8] Wong H, Hooper AWM, Niibori Y, Lee SJ, Hategan LA, Zhang L (2020). Sexually dimorphic patterns in electroencephalography power spectrum and autism-related behaviors in a rat model of Fragile X syndrome. Neurobiol Dis.

[9] Kazdoba TM, Leach PT, Silverman JL, Crawley JN (2014). Modeling Fragile X syndrome in the Fmr1 knockout mouse. Intractable Rare Dis Res.

[10] Bryda EC (2013). The Mighty Mouse: the impact of rodents on advances in biomedical research. Mo Med.

[11] Jiménez JA, Zylka MJ (2021). Controlling litter effects to enhance rigor and reproducibility with rodent models of neurodevelopmental disorders. J Neurodev Disord.

[12] Lazic SE, Essioux L (2013). Improving basic and translational science by accounting for litter-to-litter variation in animal models. BMC Neurosci.

[13] Polissidis A, Chouliara O, Galanopoulos A, Rentesi G, Dosi M, Hyphantis T (2010). Individual differences in the effects of cannabinoids on motor activity, dopaminergic activity and DARPP-32 phosphorylation in distinct regions of the brain. Int J Neuropsychopharmacol.

[14] Seibenhener ML, Wooten MC. Use of the open field maze to measure locomotor and anxiety-like behavior in mice. J Vis Exp. 2015;96:e52434. 10.3791/52434 PMC4354627 25742564

[15] Poulia N, Delis F, Brakatselos C, Lekkas P, Kokras N, Dalla C (2020). Escalating low-dose Δ9 -tetrahydrocannabinol exposure during adolescence induces differential behavioral and neurochemical effects in male and female adult rats. Eur J Neurosci.

[16] Galanopoulos A, Polissidis A, Georgiadou G, Papadopoulou-Daifoti Z, Nomikos GG, Pitsikas N (2014). WIN55,212-2 impairs non-associative recognition and spatial memory in rats via CB1 receptor stimulation. Pharm Biochem Behav.

[17] Brakatselos C, Delis F, Asprogerakas M-Z, Lekkas P, Tseti I, Tzimas PS (2021). Cannabidiol modulates the motor profile and NMDA receptor-related alterations induced by ketamine. Neuroscience.

[18] Poulia N, Delis F, Brakatselos C, Ntoulas G, Asprogerakas M-Z, Antoniou K (2021). CBD effects on motor profile and neurobiological indices related to glutamatergic function induced by repeated ketamine pre-administration. Front Pharm.

[19] Kokras N, Dioli C, Paravatou R, Sotiropoulos MG, Delis F, Antoniou K (2020). Psychoactive properties of BNN27, a novel neurosteroid derivate, in male and female rats. Psychopharmacology.

[20] Papatheodoropoulos C, Kouvaros S (2016). High-frequency stimulation-induced synaptic potentiation in dorsal and ventral CA1 hippocampal synapses: the involvement of NMDA receptors, mGluR5, and (L-type) voltage-gated calcium channels. Learn Mem.

[21] Kim D, Langmead B, Salzberg SL (2015). HISAT: a fast spliced aligner with low memory requirements. Nat Methods.

[22] Anders S, Pyl PT, Huber W (2015). HTSeq-a Python framework to work with high-throughput sequencing data. Bioinformatics.

[23] Love MI, Huber W, Anders S (2014). Moderated estimation of fold change and dispersion for RNA-seq data with DESeq2. Genome Biol.

[24] Liao Y, Wang J, Jaehnig EJ, Shi Z, Zhang B (2019). WebGestalt 2019: gene set analysis toolkit with revamped UIs and APIs. Nucleic Acids Res.

[25] Wang J, Vasaikar S, Shi Z, Greer M, Zhang B (2017). WebGestalt 2017: a more comprehensive, powerful, flexible and interactive gene set enrichment analysis toolkit. Nucleic Acids Res.

[26] Wang J, Duncan D, Shi Z, Zhang B (2013). WEB-based GEne SeT analysis toolkit (WebGestalt): update 2013. Nucleic Acids Res.

[27] Zhang B, Kirov S, Snoddy J (2005). WebGestalt: an integrated system for exploring gene sets in various biological contexts. Nucleic Acids Res.

[28] Kanehisa M, Goto S (2000). KEGG: kyoto encyclopedia of genes and genomes. Nucleic Acids Res.

[29] Kanehisa M, Furumichi M, Sato Y, Ishiguro-Watanabe M, Tanabe M (2021). KEGG: integrating viruses and cellular organisms. Nucleic Acids Res.

[30] Kanehisa M, Furumichi M, Sato Y, Kawashima M, Ishiguro-Watanabe M (2023). KEGG for taxonomy-based analysis of pathways and genomes. Nucleic Acids Res.

[31] Thiel CM, Müller CP, Huston JP, Schwarting RK (1999). High versus low reactivity to a novel environment: behavioural, pharmacological and neurochemical assessments. Neuroscience.

[32] Antunes M, Biala G (2012). The novel object recognition memory: neurobiology, test procedure, and its modifications. Cogn Process.

[33] Ennaceur A, Delacour J (1988). A new one-trial test for neurobiological studies of memory in rats. 1: Behavioral data. Behav Brain Res.

[34] Chen WS, Bear MF (2007). Activity-dependent regulation of NR2B translation contributes to metaplasticity in mouse visual cortex. Neuropharmacology.

[35] Yashiro K, Philpot BD (2008). Regulation of NMDA receptor subunit expression and its implications for LTD, LTP, and metaplasticity. Neuropharmacology.

[36] Xu T, Yu X, Perlik AJ, Tobin WF, Zweig JA, Tennant K (2009). Rapid formation and selective stabilization of synapses for enduring motor memories. Nature.

[37] Contractor A, Klyachko VA, Portera-Cailliau C (2015). Altered neuronal and circuit excitability in Fragile X syndrome. Neuron.

[38] D’Elia A, Schiavi S, Manduca A, Rava A, Buzzelli V, Ascone F (2022). FMR1 deletion in rats induces hyperactivity with no changes in striatal dopamine transporter availability. Sci Rep.

[39] Yamazaki M, Arai T, Yarimizu J, Matsumoto M (2022). 5-HT5A receptor antagonist ASP5736 ameliorates several abnormal behaviors in an Fmr1-targeted transgenic male rat model of Fragile X syndrome. Int J Neuropsychopharmacol.

[40] Kozono N, Okamura A, Honda S, Matsumoto M, Mihara T (2020). Gamma power abnormalities in a Fmr1-targeted transgenic rat model of Fragile X syndrome. Sci Rep.

[41] Hamilton SM, Green JR, Veeraragavan S, Yuva L, McCoy A, Wu Y (2014). Fmr1 and Nlgn3 knockout rats: novel tools for investigating autism spectrum disorders. Behav Neurosci.

[42] Tian Y, Yang C, Shang S, Cai Y, Deng X, Zhang J (2017). Loss of FMRP impaired hippocampal long-term plasticity and spatial learning in rats. Front Mol Neurosci.

[43] Golden CEM, Breen MS, Koro L, Sonar S, Niblo K, Browne A (2019). Deletion of the KH1 domain of Fmr1 leads to transcriptional alterations and attentional deficits in rats. Cereb Cortex.

[44] Hooper AWM, Wong H, Niibori Y, Abdoli R, Karumuthil-Melethil S, Qiao C (2021). Gene therapy using an ortholog of human Fragile X mental retardation protein partially rescues behavioral abnormalities and EEG activity. Mol Ther Methods Clin Dev.

[45] Schiavi S, Carbone E, Melancia F, di Masi A, Jarjat M, Brau F (2022). Phosphodiesterase 2A inhibition corrects the aberrant behavioral traits observed in genetic and environmental preclinical models of autism spectrum disorder. Transl Psychiatry.

[46] Schiavi S, Carbone E, Melancia F, Buzzelli V, Manduca A, Campolongo P (2022). Perinatal supplementation with omega-3 fatty acids corrects the aberrant social and cognitive traits observed in a genetic model of autism based on FMR1 deletion in rats. Nutr Neurosci.

[47] Buzzelli V, Carbone E, Manduca A, Schiavi S, Feo A, Perederiy JV (2023). Psilocybin mitigates the cognitive deficits observed in a rat model of Fragile X syndrome. Psychopharmacology.

[48] Asiminas A, Jackson AD, Louros SR, Till SM, Spano T, Dando O, et al. Sustained correction of associative learning deficits after brief, early treatment in a rat model of Fragile X syndrome. Sci Transl Med. 2019;11:eaao0498. 10.1126/scitranslmed.aao0498. 10.1126/scitranslmed.aao0498 PMC8162683 31142675

[49] Till SM, Asiminas A, Jackson AD, Katsanevaki D, Barnes SA, Osterweil EK (2015). Conserved hippocampal cellular pathophysiology but distinct behavioural deficits in a new rat model of FXS. Hum Mol Genet.

[50] Biag HMB, Potter LA, Wilkins V, Afzal S, Rosvall A, Salcedo‐Arellano MJ, et al. Metformin treatment in young children with Fragile X syndrome. Mol Genet Genomic Med. 2019;7:e956. 10.1002/mgg3.956. 10.1002/mgg3.956 PMC6825840 31520524

[51] Hustyi KM, Hall SS, Jo B, Lightbody AA, Reiss AL (2014). Longitudinal trajectories of aberrant behavior in Fragile X syndrome. Res Dev Disabil.

[52] Kashima R, Roy S, Ascano M, Martinez-Cerdeno V, Ariza-Torres J, Kim S (2016). Augmented noncanonical BMP type II receptor signaling mediates the synaptic abnormality of Fragile X syndrome. Sci Signal.

[53] Ng M-C, Yang Y-L, Lu K-T (2013). Behavioral and synaptic circuit features in a zebrafish model of Fragile X syndrome. PLoS ONE.

[54] Yu F, Zhong P, Liu X, Sun D, Gao H, Liu Q (2013). Metabotropic glutamate receptor I (mGluR1) antagonism impairs cocaine-induced conditioned place preference via inhibition of protein synthesis. Neuropsychopharmacology.

[55] Xu Z, Yang Q, Ma L, Liu S, Chen G, Wu Y (2012). Deficits in LTP induction by 5-HT2A receptor antagonist in a mouse model for Fragile X syndrome. PLoS ONE.

[56] Bostrom CA, Majaess N-M, Morch K, White E, Eadie BD, Christie BR (2015). Rescue of NMDAR-dependent synaptic plasticity in Fmr1 knock-out mice. Cereb Cortex.

[57] Toft AKH, Lundbye CJ, Banke TG (2016). Dysregulated NMDA-receptor signaling inhibits long-term depression in a mouse model of Fragile X syndrome. J Neurosci.

[58] Lundbye CJ, Toft AKH, Banke TG (2018). Inhibition of GluN2A NMDA receptors ameliorates synaptic plasticity deficits in the Fmr1-/y mouse model. J Physiol.

[59] Darnell JC, Klann E (2013). The translation of translational control by FMRP: therapeutic targets for FXS. Nat Neurosci.

[60] Bassell GJ, Warren ST (2008). Fragile X syndrome: loss of local mRNA regulation alters synaptic development and function. Neuron.

[61] Derkach VA, Oh MC, Guire ES, Soderling TR (2007). Regulatory mechanisms of AMPA receptors in synaptic plasticity. Nat Rev Neurosci.

[62] Thiagarajan TC, Lindskog M, Tsien RW (2005). Adaptation to synaptic inactivity in hippocampal neurons. Neuron.

[63] Clem RL, Barth A (2006). Pathway-specific trafficking of native AMPARs by in vivo experience. Neuron.

[64] Cull-Candy S, Kelly L, Farrant M (2006). Regulation of Ca2+-permeable AMPA receptors: synaptic plasticity and beyond. Curr Opin Neurobiol.

[65] Chatterjee M, Kurup PK, Lundbye CJ, Hugger Toft AK, Kwon J, Benedict J (2018). STEP inhibition reverses behavioral, electrophysiologic, and synaptic abnormalities in Fmr1 KO mice. Neuropharmacology.

[66] Davidovic L, Navratil V, Bonaccorso CM, Catania MV, Bardoni B, Dumas M-E (2011). A metabolomic and systems biology perspective on the brain of the Fragile X syndrome mouse model. Genome Res.

[67] Hertz L, Schousboe A. Primary cultures of gabaergic and glutamatergic neurons as model systems to study neurotransmitter functions I. Differentiated Cells. In: Model systems of development and aging of the nervous system. Boston, MA: Springer USA; 1987. p. 19–31.

[68] Kalmbach BE, Johnston D, Brager DH (2015). Cell-type specific channelopathies in the prefrontal cortex of the *fmr1-/y* mouse model of Fragile X syndrome. eNeuro.

[69] Luque MA, Beltran-Matas P, Marin MC, Torres B, Herrero L (2017). Excitability is increased in hippocampal CA1 pyramidal cells of Fmr1 knockout mice. PLoS ONE.

[70] Deng P-Y, Carlin D, Oh YM, Myrick LK, Warren ST, Cavalli V (2019). Voltage-independent SK-channel dysfunction causes neuronal hyperexcitability in the hippocampus of *Fmr1* knock-out mice. J Neurosci.

[71] Booker SA, Simões de Oliveira L, Anstey NJ, Kozic Z, Dando OR, Jackson AD (2020). Input-output relationship of CA1 pyramidal neurons reveals intact homeostatic mechanisms in a mouse model of Fragile X syndrome. Cell Rep.

[72] Strange BA, Witter MP, Lein ES, Moser EI (2014). Functional organization of the hippocampal longitudinal axis. Nat Rev Neurosci.

[73] Gulyaeva NV (2019). Functional neurochemistry of the ventral and dorsal hippocampus: stress, depression, dementia and remote hippocampal damage. Neurochem Res.

[74] Papatheodoropoulos C (2018). Electrophysiological evidence for long-axis intrinsic diversification of the hippocampus. Front Biosci.

[75] Haussler U, Bielefeld L, Froriep UP, Wolfart J, Haas CA (2012). Septotemporal position in the hippocampal formation determines epileptic and neurogenic activity in temporal lobe epilepsy. Cereb Cortex.

[76] Papatheodoropoulos C, Moschovos C, Kostopoulos G (2005). Greater contribution of N-methyl-d-aspartic acid receptors in ventral compared to dorsal hippocampal slices in the expression and long-term maintenance of epileptiform activity. Neuroscience.

[77] Rao JS, Kellom M, Kim H-W, Rapoport SI, Reese EA (2012). Neuroinflammation and synaptic loss. Neurochem Res.

[78] Citri A, Malenka RC (2008). Synaptic plasticity: multiple forms, functions, and mechanisms. Neuropsychopharmacology.

[79] Peters KZ, Cheer JF, Tonini R (2021). Modulating the neuromodulators: dopamine, serotonin, and the endocannabinoid system. Trends Neurosci.

[80] Buddell T, Quinn CC. An autism-associated calcium channel variant causes defects in neuronal polarity in the ALM neuron of *C. elegans*. MicroPubl Biol. 2021;2021. 10.17912/micropub.biology.000378. 10.17912/micropub.biology.000378 PMC8017444 33829152

[81] Liu X, Kumar V, Tsai N-P, Auerbach BD. Hyperexcitability and homeostasis in Fragile X syndrome. Front Mol Neurosci. 2022;14:805929. 10.3389/fnmol.2021.805929. 10.3389/fnmol.2021.805929 PMC8770333 35069112

[82] Duffney LJ, Wei J, Cheng J, Liu W, Smith KR, Kittler JT (2013). Shank3 deficiency induces NMDA receptor hypofunction via an actin-dependent mechanism. J Neurosci.

[83] Lee K, Vyas Y, Garner CC, Montgomery JM (2019). Autism‐associated *Shank3* mutations alter mGluR expression and mGluR‐dependent but not NMDA receptor‐dependent long‐term depression. Synapse.

[84] Uchino S, Waga C (2013). SHANK3 as an autism spectrum disorder-associated gene. Brain Dev.

[85] Bhat S, Dao DT, Terrillion CE, Arad M, Smith RJ, Soldatov NM (2012). CACNA1C (Cav1.2) in the pathophysiology of psychiatric disease. Prog Neurobiol.

